# Supplementary material for: ORP5 and ORP8 bind phosphatidylinositol-4, 5-biphosphate (PtdIns(4,5)P2) and regulate its level at the plasma membrane
Source: Nat Commun. 2017 Oct 2;8:757. doi: 10.1038/s41467-017-00861-5 (PMC5624964; doi:10.1038/s41467-017-00861-5)
Supplement: Supplementary file 1 — Supplementary Information [file 41467_2017_861_MOESM1_ESM.pdf]

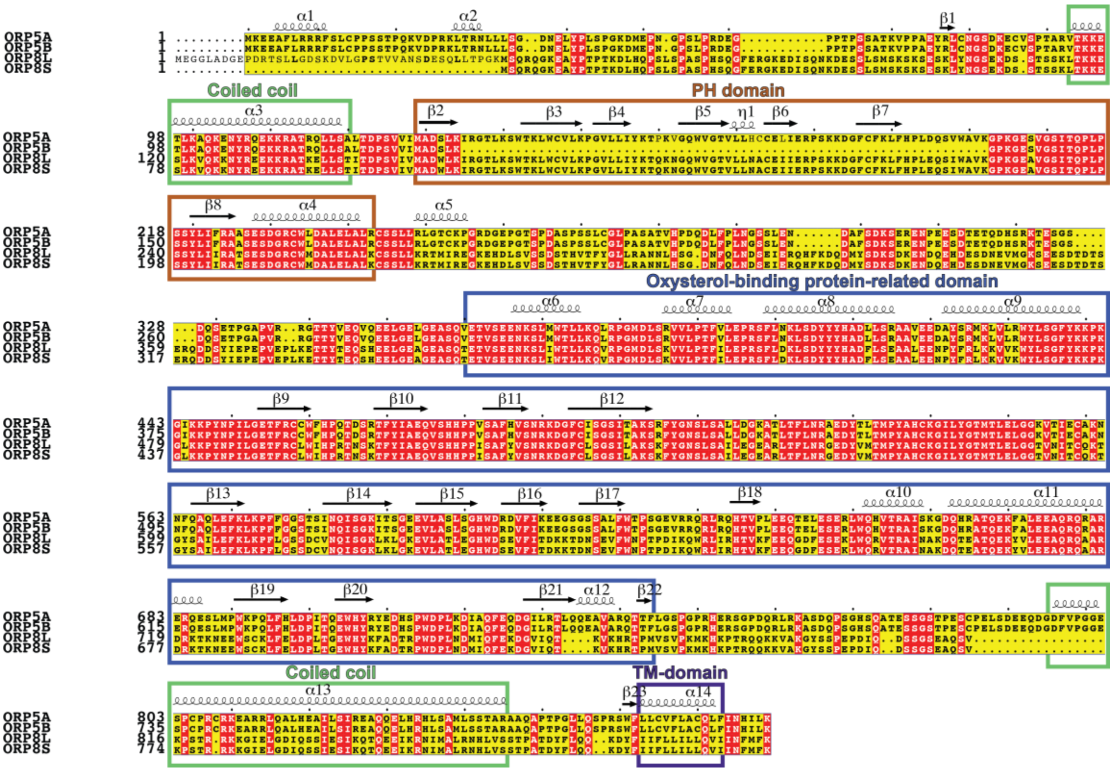

Supplementary Figure 1. Sequence alignment of the human ORP5 and ORP8 proteins. Sequences are shown for the human ORP5 and ORP8 splice variants. Colored boxes represent the individual domains.

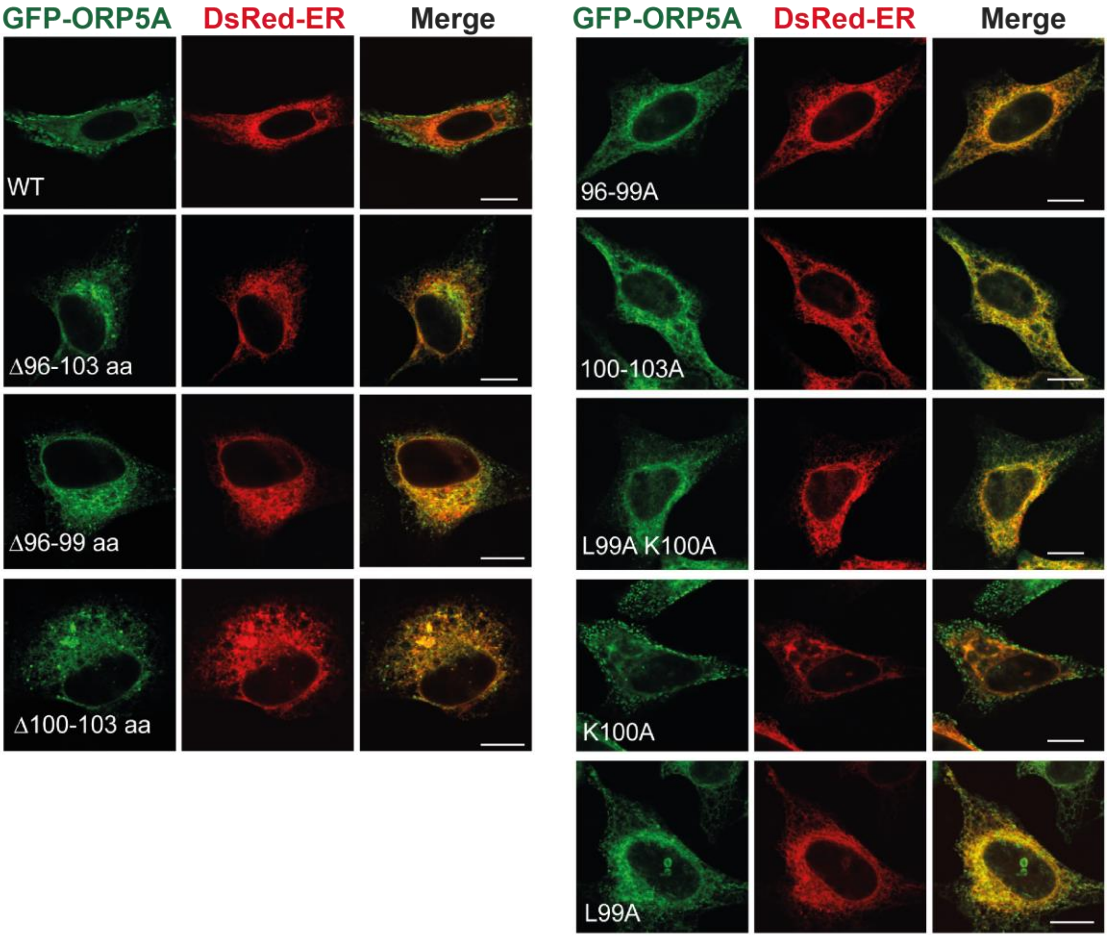

Supplementary Figure 2. Truncation and mutagenesis analyses of ORP5 PM tetherting by fluorescence microscopy. Various truncated and mutant ORP5A constructs were tested for their targeting to the PM using confocal microscopy. Bar = 10 μm

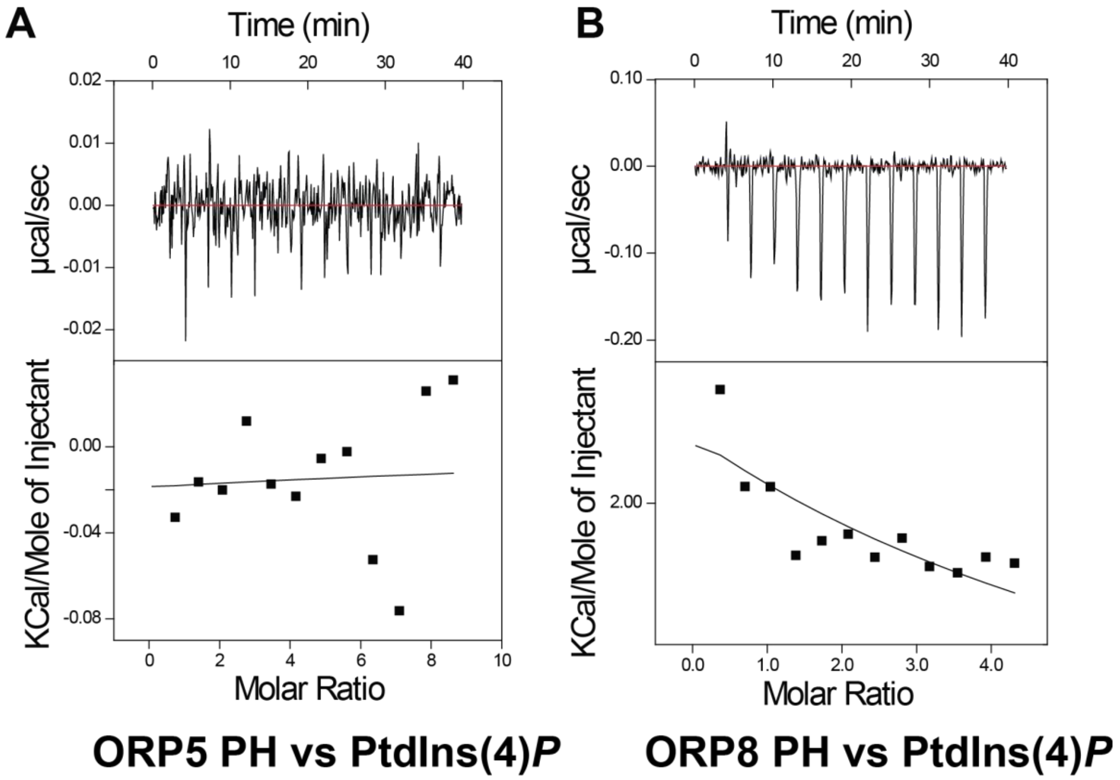

Supplementary Figure 3. ORP5 and ORP8 PH domain do not bind PtdIns(4)*P*.

(**A, B**) The binding of ORP5 and ORP8 PH domain to PtdIns(4)*P* was measured by ITC. No binding signal was observed. Experiments were performed at 25°C using 25 μM protein and 500 μM PtdIns(4)*P*. Top panels show raw data and bottom panels show integrated normalised data.

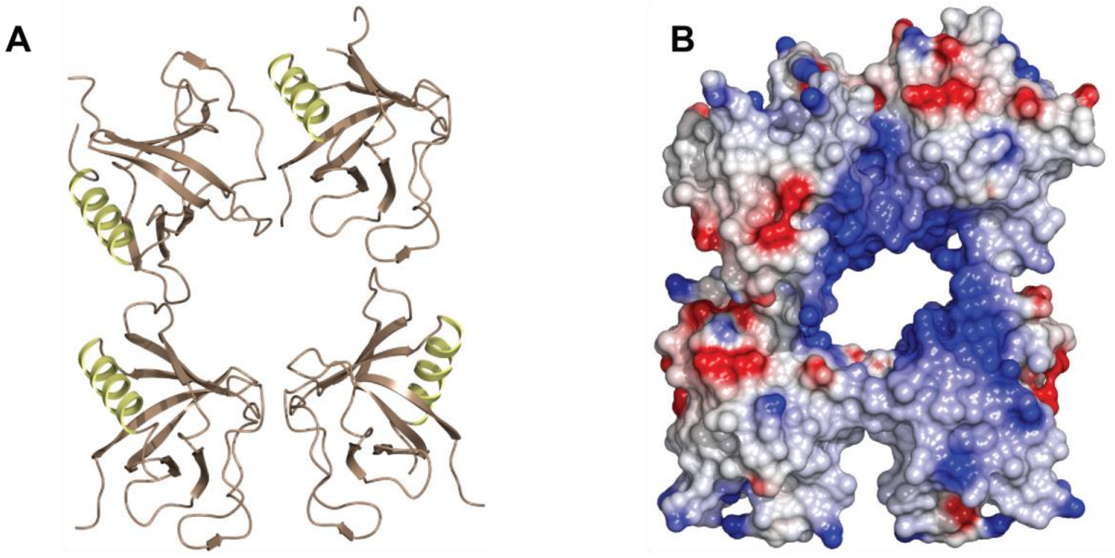

ORP8 PH (crystallised in the presence of IP6)

Supplementary Figure 4. Crystal structure of ORP8 PH crystallized in the presence of inositol hexaphosphate. (**A**) Ribbon representation of the asymmetric unit of ORP8 PH domain with four polypeptide chains. There are four identical ORP8 PH domain chains in the asymmetric unit. (**B**) Electrostatic potential surface representation of the asymmetric unit of ORP8 PH domain. Electrostatic potential rendered surface was computed in ccp4mg [1], negatively charged surfaces are shown in red whereas positively charged surfaces are blue in colour, colours are contoured from -0.5 eV to +0.5 eV.

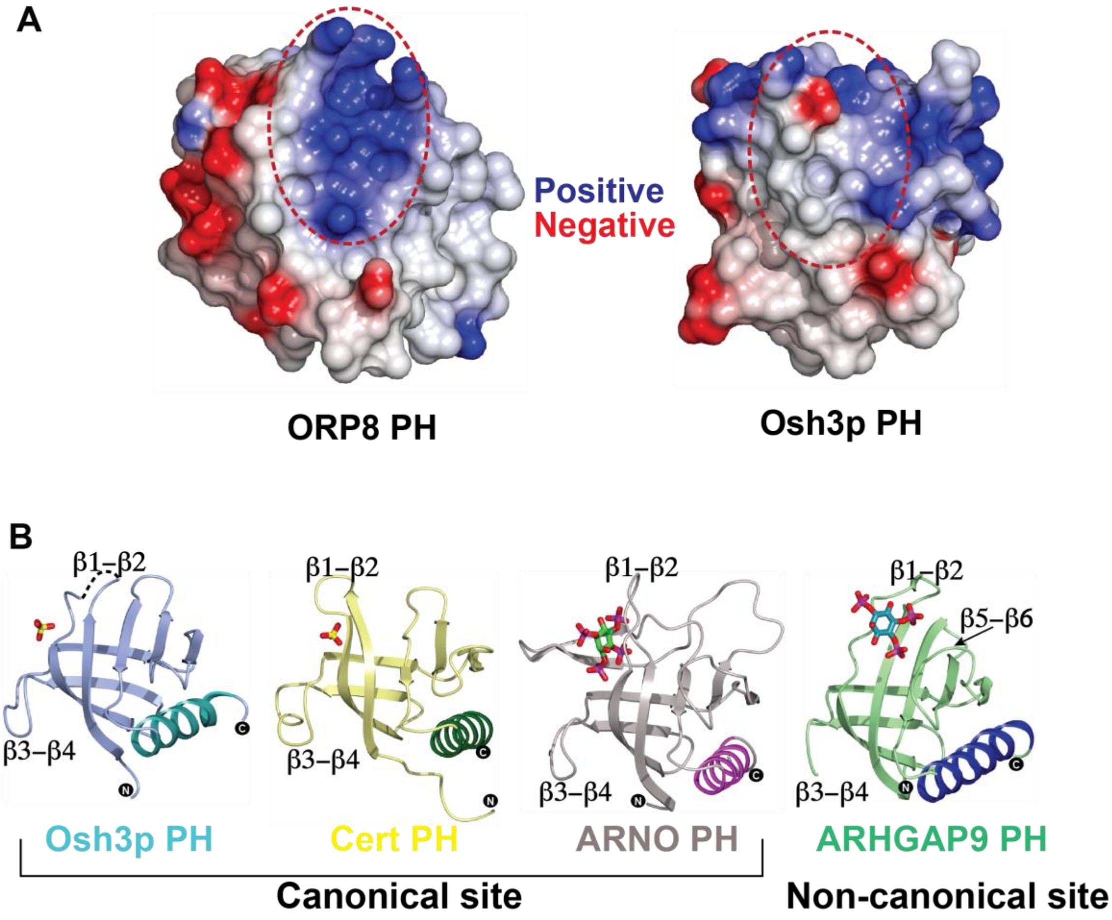

Supplementary Figure 5. Canonical and non-canonical PtdIns*P* binding modes of PH domains. (A) Comparison of the electrostatic surface of ORP8 PH and Osh3p PH domain highlights a basic patch (putative lipid binding region) that is missing in Osh3p PH indicating that the ORP8 PH domain adopts a non-canonical lipid-binding regime. Electrostatic potential rendered surface was computed in ccp4mg [1], negatively charged surfaces are shown in red whereas positively charged surfaces are blue in colour, colours are contoured from -0.5 eV to +0.5 eV. (**B**) Osh3p, Cert and ARNO PH domain bind PtdIns*P* at the canonical site that is between the β1- β2 and β3- β4 loop. ARHGAP9 PH domain binds PtdIns*P* at the non-canonical site (between the β1- β2 and β5- β6 loops). All the structures were superimposed on each other and are shown in the same orientation.

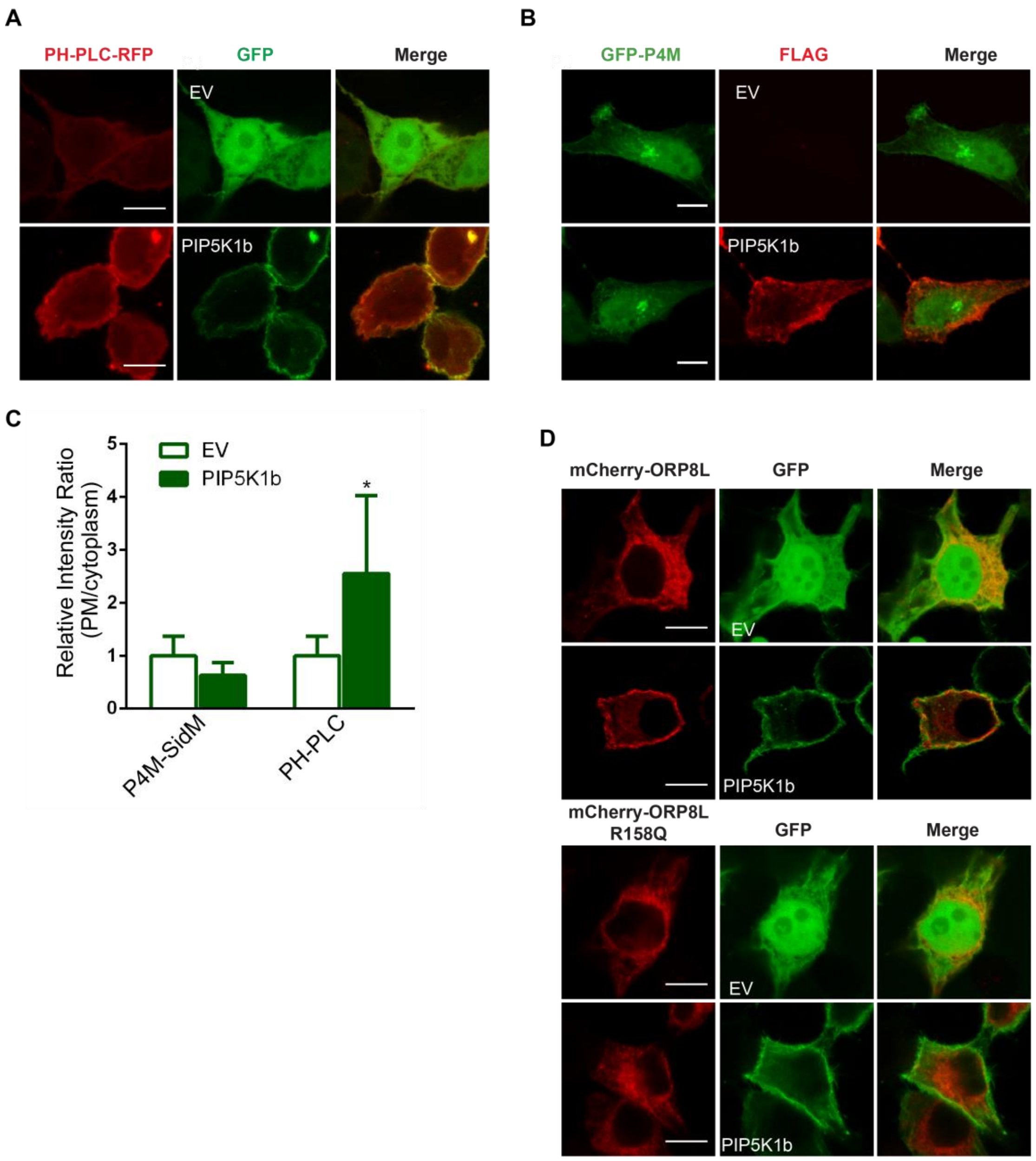

Supplementary Figure 6. Effect of PIP5K1b overexpression on ORP8L localization. (**A**) Co-expression of PH-PLC-RFP with GFP empty vector (EV) or GFP-PIP5K1b in HeLa cells. (**B**) Co-expression of GFP-P4M with FLAG-PIP5K1b in HeLa cells. (**C**) Quantitation of PM/Cytoplasm intensity ratio of PH-PLC-RFP in (A) and GFP-P4M in (B). (**D**) Co-expression of mCherry-ORP8L or -ORP8L R158Q with GFP EV or GFP-PIP5K1b (mean + SD; $*P < 0.05$, 2way ANOVA, n = 5 ~ 9 cells). Bar = 10 μm.

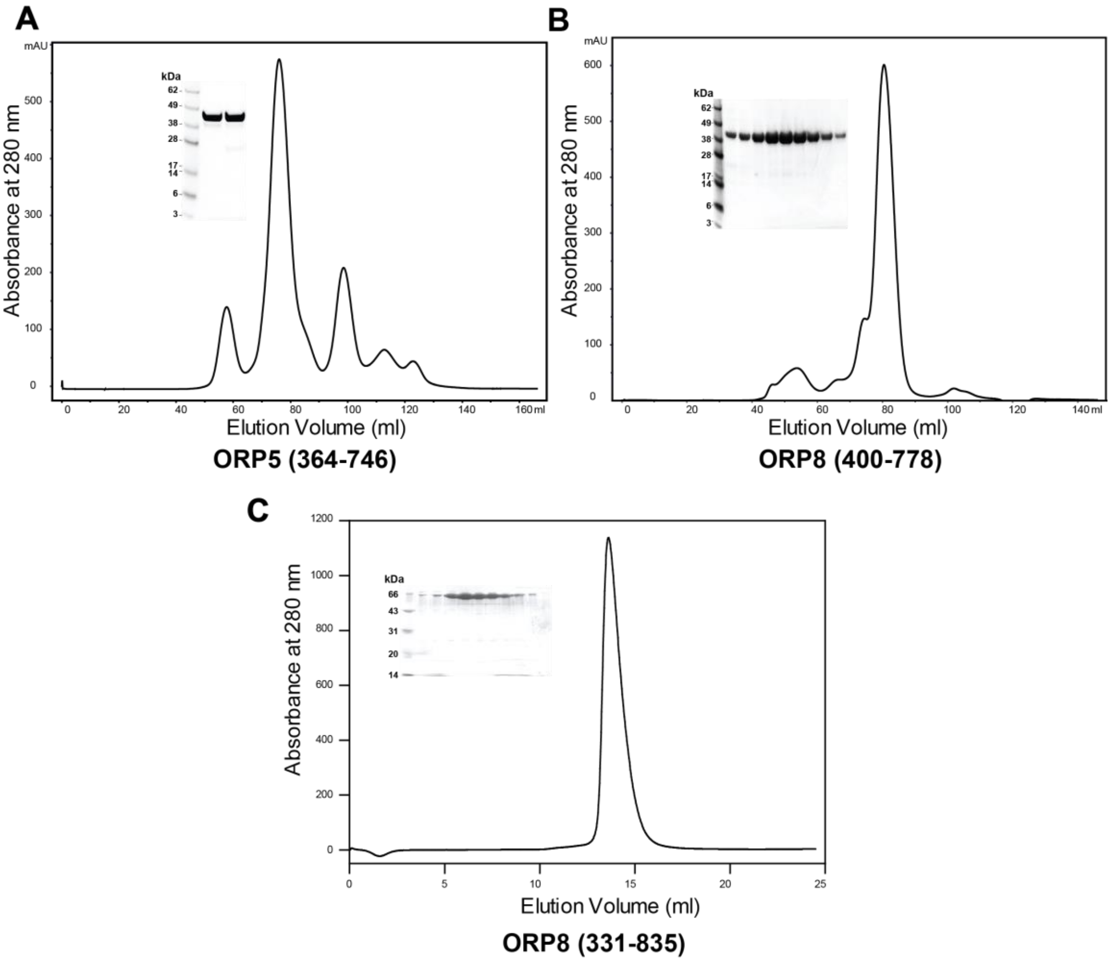

Supplementary Figure 7. Purification of ORD5 and ORD8 domain. (**A, B, C**) Chromatograms and SDS-PAGE gel showing highly pure and monodisperse ORP5 (364-746), ORP8 (400-778) and ORP8 (331-835) purified recombinantly from E.*coli*. ORP5 (364-746) and ORP8 (400-778) was resolved on superdex200 16/60 whereas ORP8 (331-835) was purified on superdex 200 10/300 size exclusion chromatography column. These proteins were used for biochemical experiments.

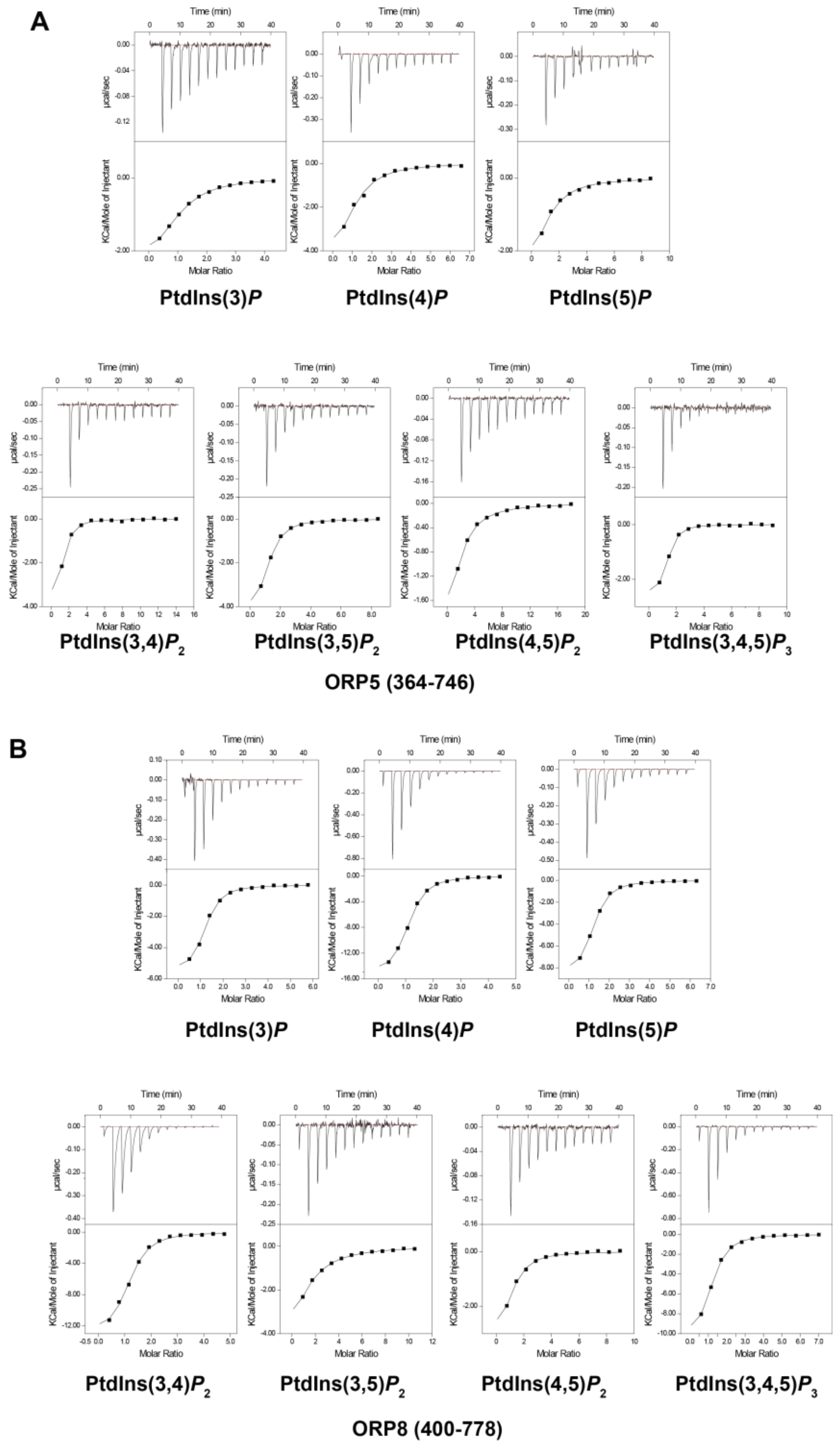

Supplementary Figure 8. ORD8/5 associates with most of the PtdIns*P* species. The binding of purified (**A**) ORD5 and (**B**) ORD8 to water-soluble PtdIns*P* species was measured by ITC. These ORD domains binds to all the PtdIns*P*s including PtdIns(4)*P*. Experiments were performed at 25˚C using 25 µM protein in the cell and 500 µM PtdIns*P*s injected from the syringe. Top panels show raw data, and bottom panels show integrated normalized data.

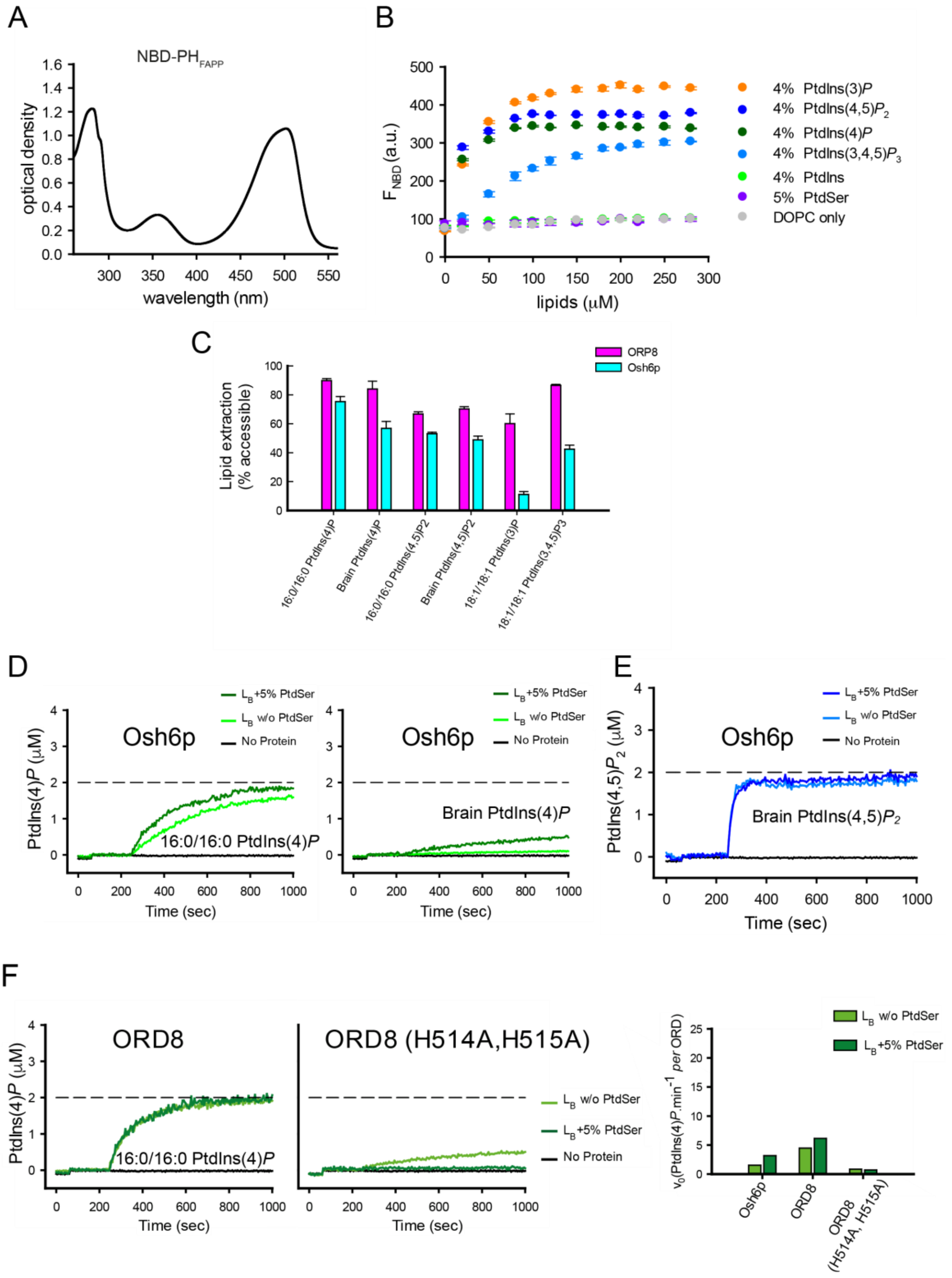

Supplementary Figure 9. Validation of PtdIns*P* sensor and the ability of Osh6p and ORP8 to transport PtdIns*P*s. (**A**) Emission spectra of NBD-PH$_{FAPP}$. (**B**) NBD-PH$_{FAPP}$ selectively recognizes all the PtdIns*P*s except PtdIns demonstrating that NBD-PH$_{FAPP}$ is a sensor for other PtdIns*P*s including PtdIns*(4)P*. PtdIns(4)*P* and

PtdIns(4,5)P_2 are from brain, the rest are 18:1/18:1. (**C**) Bar graph showing the percentage of PtdIns*P* with varying acyl chain as well as brain PtdIns*P*s extracted from liposomes by Osh6p, ORD8. Error bars indicate s.d.; n = 3 (**D, E**) Transport of C16:0/C16:0 PtdIns(4)*P*, brain PtdIns(4)*P*, and brain PtdIns(4,5)P_2 by Osh6p to liposomes containing 5% PtdSer. **(F).** PtdIns(4)*P* (C16:0/C16:0) transport assay by ORD8 and its H514A/H515A mutant. Donor liposomes were incubated with NBD-PH_{FAPP} followed by addition of acceptor liposomes doped with or without PtdSer. After 3 min, the protein was injected. The broken line signifies NBD-PH_{FAPP} signal upon complete PtdIns(4)*P* equilibration between liposomes. The plot (right) shows initial PtdIns(4)*P* transport rates. Addition of 5% PtdSer only slightly increased the transfer rates.

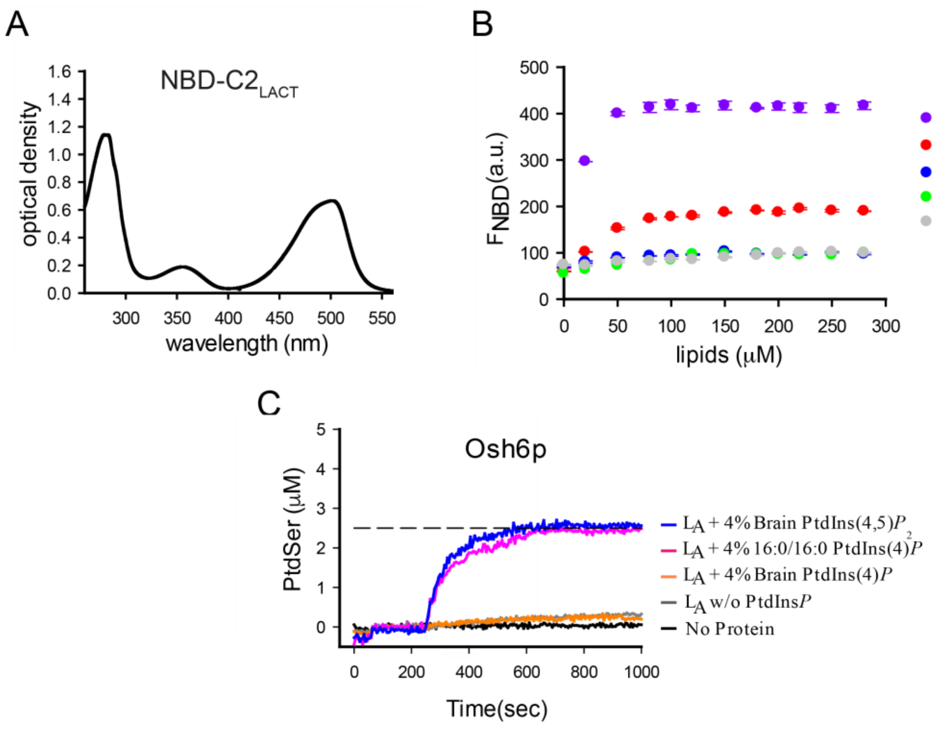

Supplementary Figure 10. Validation of Lactadherin-C2 domain as PtdSer sensor and Osh6p's ability to transport PtdSer in a PtdIns*P* gradient. (**A**) Emission spectra of NBD-C2$_{LACT}$. (**B**) NBD-C2$_{Lact}$ selectively binds to liposomes containing PtdSer. (**C**) Osh6p transports PtdSer under a brain PtdIns(4,5)P_2 as well as a PtdIns(4)*P* (C16:0/C16:0) gradient. The PtdSer transfer was barely detectable under a brain PtdIns(4)*P* gradient. In (C), brain PtdIns(4)*P*, PtdIns(4)*P* (C16:0/C16:0), brain PtdIns(4,5)P_2 and PtdSer were used in liposome B as indicated in Figure 6A.

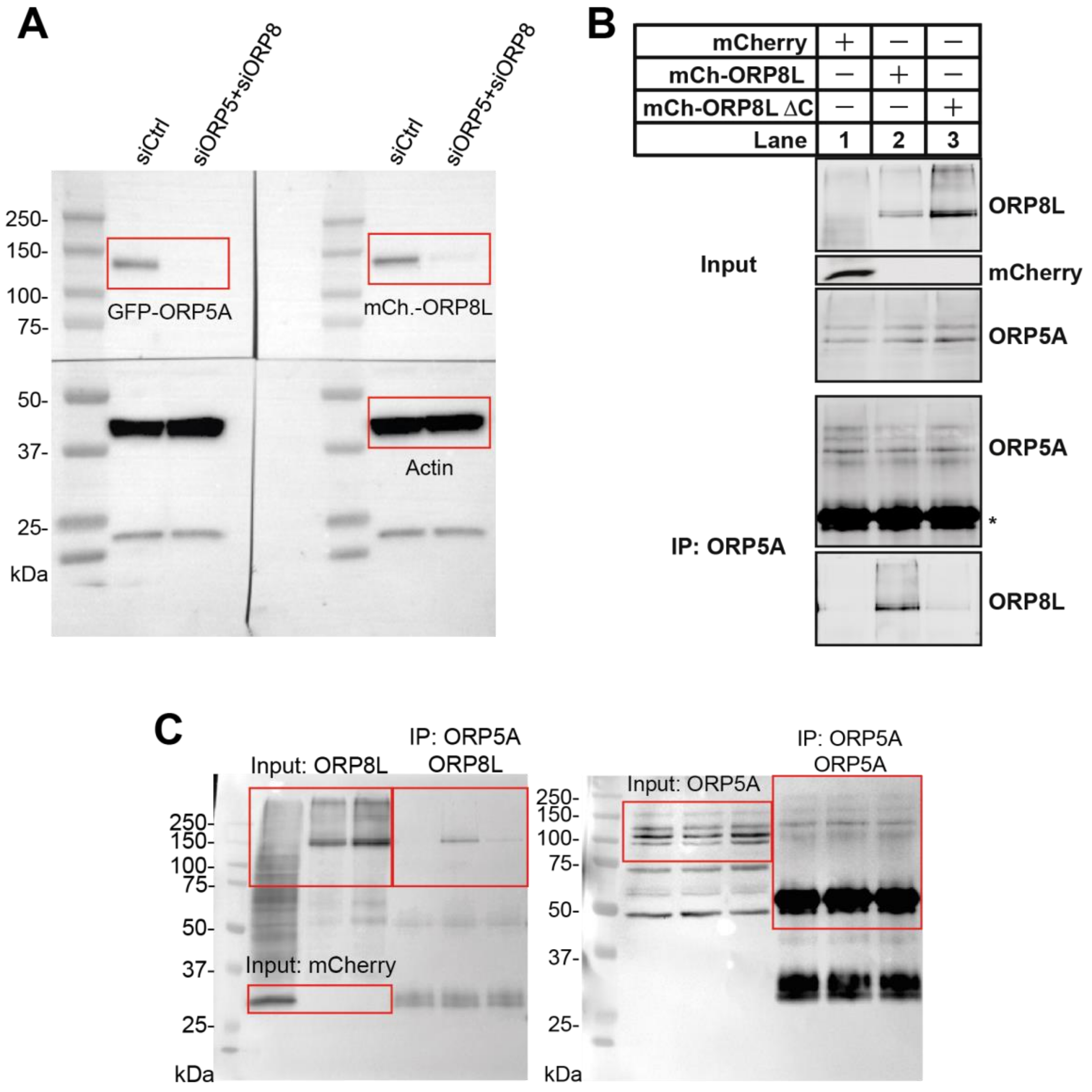

Supplementary Figure 11. ORP5 physically interacts with ORP8. (**A**) Unprocessed scan of western blot shown in figure 5G. (**B, C**) Immunoprecipitation showing ORP5A can associate with mCherry tagged ORP8L. Loss of the transmembrane domain (ORP8LΔC) significantly reduces the interaction indicating the two proteins associate at the ER membrane.

A

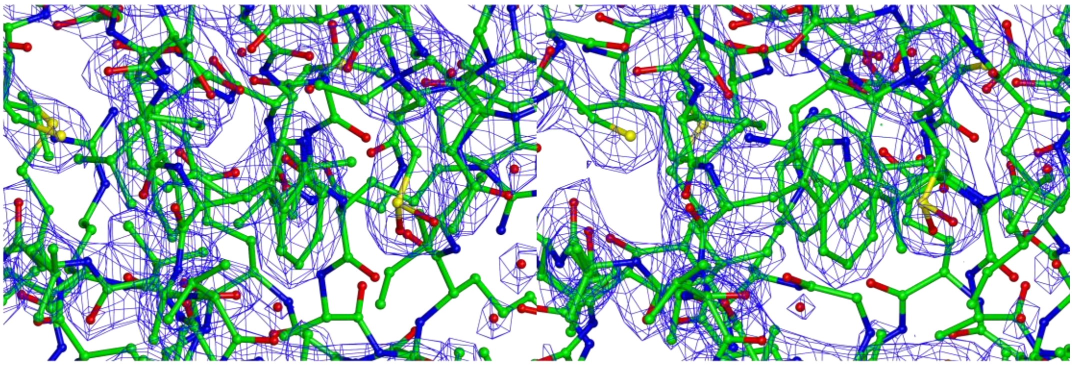

B

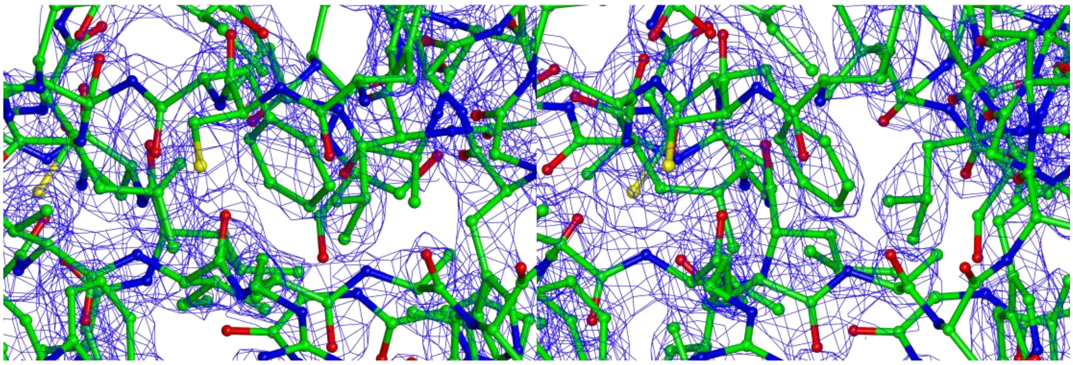

Supplementary Figure 12. Stereo image showing well defined F_o-F_c electron density map contoured at 3σ. (A) ORP8 PH domain crystallised in P6522 space group. (B) ORP8 PH domain crystallised in P1211 space group.

SUPPLEMENTARY REFERENCES

1. McNicholas, S., Potterton, E., Wilson, K.S. & Noble, M.E. Presenting your structures: the CCP4mg molecular-graphics software. *Acta Crystallogr D Biol Crystallogr* **67**, 386-394 (2011).
